# Estimated Short-Term Effects of Coarse Particles on Daily Mortality in Stockholm, Sweden

**DOI:** 10.1289/ehp.1103995

**Published:** 2011-12-19

**Authors:** Kadri Meister, Christer Johansson, Bertil Forsberg

**Affiliations:** 1 Department of Public Health and Clinical Medicine, Occupational and Environmental Medicine, Umeå University, Umeå, Sweden; 2 Department of Applied Environmental Science, Stockholm University, Stockholm, Sweden; 3 Environment and Health Administration, Stockholm, Sweden

**Keywords:** coarse particles, health effects, mortality, PM_2.5_, PM_10_, road dust, short-term exposure

## Abstract

Background: Although serious health effects associated with particulate matter (PM) with aerodynamic diameter ≤ 10 μm (PM_10_) and ≤ 2.5 μm (PM_2.5_; fine fraction) are documented in many studies, the effects of coarse PM (PM_2.5–10_) are still under debate.

Objective: In this study, we estimated the effects of short-term exposure of PM_2.5–10_ on daily mortality in Stockholm, Sweden.

Method: We collected data on daily mortality for the years 2000 through 2008. Concentrations of PM_10_, PM_2.5_, ozone, and carbon monoxide were measured simultaneously in central Stockholm. We used additive Poisson regression models to examine the association between daily mortality and PM_2.5–10_ on the day of death and the day before. Effect estimates were adjusted for other pollutants (two-pollutant models) during different seasons.

Results: We estimated a 1.68% increase [95% confidence interval (CI): 0.20%, 3.15%] in daily mortality per 10-μg/m^3^ increase in PM_2.5–10_ (single-pollutant model). The association with PM_2.5–10_ was stronger for November through May, when road dust is most important (1.69% increase; 95% CI: 0.21%, 3.17%), compared with the rest of the year (1.31% increase; 95% CI: –2.08%, 4.70%), although the difference was not statistically significant. When adjusted for other pollutants, particularly PM_2.5_, the effect estimates per 10 μg/m^3^ for PM_2.5–10_ decreased slightly but were still higher than corresponding effect estimates for PM_2.5_.

Conclusions: Our analysis shows an increase in daily mortality associated with elevated urban background levels of PM_2.5–10_. Regulation of PM_2.5–10_ should be considered, along with actions to specifically reduce PM_2.5–10_ emissions, especially road dust suspension, in cities.

*Particle effects on mortality.* Hundreds of epidemiological studies have shown that the ambient particulate air pollution is associated with daily mortality, generally studied using the concentration of particulate matter (PM) with an aerodynamic diameter ≤ 10 μm (PM_10_) or fine PM with aerodynamic diameter of ≤ 2.5 μm (PM_2.5_) (Samoli et al. 2008). The effect of coarse PM (PM_2.5–10_) on mortality has been less studied. In their review article, Brunekreef and Forsberg (2005) concluded that most published mortality studies that applied two-pollutant models were unable to demonstrate independent PM_2.5–10_ effects on mortality after adjusting for PM_2.5_. However, PM_2.5–10_ levels are expected to be more spatially heterogeneous than are PM_2.5_ levels, which increase exposure misclassification when one or a few monitors provide exposure data (Monn 2001). Moreover, most time-series studies that have reported significant effects on mortality associated with PM_2.5–10_ were conducted in arid areas, including such places as Phoenix, Arizona (Mar et al. 2000), Coachella Valley, California (Ostro et al. 2000), and Mexico City (Castillejos et al. 2000). In arid areas, particle dust often originates from the surrounding land, not from local point sources, and particle levels are therefore expected to be more spatially homogeneous.

In a more recent study, Malig and Ostro (2009) used data from 15 counties in California and found an association between PM_2.5–10_ and daily mortality (both all-cause and cardiovascular mortality), particularly among demographic subgroups of lower socioeconomic status. In their study, only those participants who resided close to an air pollution monitor were included in the study in order to reduce exposure misclassification. Adjusting for PM_2.5_ had no effect on the effect estimates for PM_2.5–10_, likely due to its low correlation with PM_2.5–10_ in these California counties. An even larger study of U.S. cities found an association between PM_2.5–10_ and daily mortality that persisted after adjusting for PM_2.5_ (Zanobetti and Schwartz 2009). Recent studies from southern Europe have explored the effects of windblown Saharan dust, including a study conducted in Barcelona, Spain, that found evidence of an effect of PM_2.5–10_ on daily mortality during Saharan dust days, despite rather moderate particle concentrations (Perez et al. 2008).

European toxicological studies have indicated that PM_2.5–10_ has the same toxicological potential as PM_2.5_ on a mass basis (Gerlofs-Nijland et al. 2007; Sandström et al. 2005). It also has been suggested that particles of crustal origin are associated with markers of inflammation and acute toxicity in bioassays (Steerenberg et al. 2006). A cluster of European *in vitro* studies have shown that for mineral particles the composition and surface reactivity appeared to be most important for the proinflammatory potential of the particles (Schwarze et al. 2007).

*PM_2.5–10_ sources and its importance for PM_10_.* A directive from the European Union (EU; European Commission 2008) regulates the total mass of all PM_10_ irrespective of size, morphology, chemistry, and health effects. In the urban environment, different sources contribute differently to total PM_10_ because of variation in the size distribution of the emitted particles (Johansson et al. 2007). At roadside locations, most traffic exhaust particles are 10–30 nm in diameter, which is too small to result in a large aerosol mass, even when number concentrations are high (Gidhagen et al. 2004). Samples collected in Berlin showed that about 45% of local traffic contributions to roadside PM_10_ concentrations were due to suspended soil material, and the remaining traffic contribution was due to vehicle exhaust and tire abrasion (Lenschow et al. 2001). Likewise, about 50% of PM_10_ during summer months in Birmingham, United Kingdom, was due to PM_2.5–10_ (Harrison et al. 1997). In northern Europe, PM_2.5–10_ concentrations are generally elevated during winter and spring because of the use of studded tires, road salt, and traction sand. In Stockholm, road wear increases drastically because of the use of studded winter tires and traction sand on streets, such that up to 90% of the locally emitted PM_10_ during the winter may be due to road abrasion (Johansson et al. 2007). Suspension of road dust is a major contributor to PM_2.5–10_ and to the exceedances of the EU limit values for PM_10_ in Stockholm (Norman and Johansson 2006).

The aim of this study was to assess the effect of PM_2.5–10_ on daily mortality in Stockholm.

## Material and Methods

*Health data.* This study of the greater Stockholm area (population ~ 1.3 million) was based on daily counts of deaths excluding external causes [*International Classification of Diseases*, *10th Revision* (ICD-10) codes A00 through R99; World Health Organization 2007], for the years 2000 through 2008 from the Cause of Death Register at the Swedish National Board of Health and Welfare (Stockholm, Sweden).

*Environmental data.* Data on daily urban background concentrations of PM_10_, PM_2.5_, ozone (O_3_), and carbon monoxide (CO) were obtained from the Environment and Health Administration of Stockholm (2011). PM_10_, PM_2.5_, and O_3_ were measured in central Stockholm at Torkel Knutssonsgatan, a monitoring station located at rooftop level (at a height of 25 m) not directly affected by nearby emissions (Johansson et al. 2007). Measurements from the same monitoring station have been used to represent fluctuations in particle and O_3_ levels in Stockholm in previous studies, such as APHEA 2 (Air Pollution and Health: A European Approach; Gryparis et al. 2004; Le Tertre et al. 2002).

The mass concentrations of PM_10_ and PM_2.5_ were measured using tapered element oscillating microbalance (TEOM 14001; Thermo Fisher Scientific, East Greenbush, NY, USA). To account for losses of volatile material in the PM, all data were corrected following Areskoug (2007). Continuous measurement of O_3_ was based on its absorption of ultraviolet light (UV Absorption Ozone Analyzer, model 42M; Environnement S.A, Poissy, France). The urban background CO concentrations were based on continuous measurements of two rooftop stations (Hornsgatan and Sveavägen, both at a height of 25 m) in central Stockholm. The instruments were based on a nondispersive infrared technique (Carbon Monoxide Analyzer, model 48; Thermo Environmental Instrument Inc., Franklin, MA, USA). The coarse fraction of PM_10_ (PM_2.5–10_) is based on the difference between PM_10_ and PM_2.5_.

The contribution of road dust to the particle concentrations varies with the wetness of the road surfaces (Norman and Johansson 2006) and is not correlated with exhaust particles (Johansson et al. 2007). The number of days with high PM_2.5–10_ levels therefore depends on the meteorological conditions, especially during the late winter and spring. Therefore, we adjusted for meterological data that was collected from the Swedish Meterological and Hydrological Institute. Daily temperature and relative humidity were measured at Bromma Airport, a city airport, 9 km from Stockholm city center.

*Statistical methods.* We studied the association between daily mortality and PM_2.5–10_ concentrations averaged over the day of death and the day before death (lag01) with a time-series analysis. The exposure lag01 has been commonly used when effects of air pollution on mortality have been studied (Gryparis et al. 2004; Katsouyanni et al. 2001; Samoli et al. 2007). Time-series analysis allows estimation of relatively small acute effects in large study populations.

We applied additive Poisson regression models, controlling for long-term trend using a smooth function with eight degrees of freedom per year, and for day of the week and public holidays using indicator variables. We controlled for the effect of weather by adjusting for the current day’s temperature and relative humidity, together with smooth functions of mean temperature and relative humidity over the previous 2 days (each using six degrees of freedom). Influenza episodes were controlled by modeling the daily influenza hospital admissions as a smooth function. All influenza hospital admissions in Sweden were obtained from the Patient Register at the Swedish National Board of Health and Welfare (Stockholm, Sweden). Each of the smooth functions in the model was represented using penalized regression splines.

We modeled 24-hr average concentrations of PM_2.5–10_, PM_2.5_, and CO and the maximum of 8-hr moving-average (between 0600 hours and 2200 hours) concentrations of O_3_ on the same day and the previous day (lag01). Results are reported for single-pollutant models (adjusted for time trend, day of the week, public holidays, temperature, humidity, and influenza outbreaks) and for multipollutant models (including two pollutants in the same model, in addition to the covariates listed above). Results also are reported for a 10-μg/m^3^ increase as well as for an interquartile range (IQR) increase in each pollutant.

The analysis was stratified by period because the composition of particles varies seasonally. In Sweden, passenger cars, light-weight trucks, and light-weight buses are required to have winter tires from 1 December to 31 March. Heavy vehicles are not required to use winter tires. Winter tires can be studded or nonstudded, but studded winter tires were allowed from 1 October to 30 April during the study period 2000 through 2008 and were used by 70–75% of vehicles in Stockholm during those years. The share of studded winter tires usually increases from zero in September through October to its maximum in December through March and then falls back to zero in May (Norman and Johansson 2006), depending on weather and road conditions. Although studded tires are banned after 1 May, road dust remains elevated because of the accumulation of PM on the road surface during the winter (Ketzel et al. 2007; Norman and Johansson 2006). Therefore, we selected November through May as the period of interest for effects of studded tires (“road dust period,” which results in in high concentrations of PM_2.5–10_) and June through October as the reference season.

To test the hypothesis that PM_2.5–10_ may affect mortality with a longer lag than lag01, we also fitted a distributed lag model for up to 7 days (same day and up to 6 days earlier) for PM_2.5–10_.

We used *p*-values < 0.05 to define statistically significant effect estimates and *p*-values between 0.05 and 0.10 to define borderline significant effect estimates. Data were analyzed using the mgcv package in R (version 2.11.1; R Core Development Team 2010).

## Results

There were 93,398 deaths (excluding deaths due to external causes) during the 2000 through 2008 study period (3,285 days). On average, there were 28.4 deaths per day (range, 12–52). The average number of deaths per day for the road dust period (November through May) was 29.6 (range, 13–52), and for the reference season (June through October) was 26.8 (range, 12–50). Days with missing values for environmental variables during the study period were not included, most often because of missing PM_2.5_ (260 days) and PM_10_ data (106 days) that did not allow calculation of PM_2.5–10_. In total, 2,789 days were included in the analysis. A statistical summary of the air pollution measurements for the whole period (2000 through 2008) is presented in Table 1.

**Table 1:** Summary of daily air pollution and meteorological data.

Variable	Season	Mean ± SD	IQR	Maximum
PM_10_ (μg/m^3^)		Overall		15.5 ± 9.6		9.4		95.2
		Nov–May		17.0 ± 10.8		11.9		95.2
		Jun–Oct		13.5 ± 7.0		6.4		67.0
PM_2.5_ (μg/m^3^)		Overall		8.6 ± 5.7		4.9		46.2
		Nov–May		8.9 ± 6.1		5.3		46.2
		Jun–Oct		8.2 ± 5.0		4.5		43.0
PM_2.5–10_ (μg/m^3^)		Overall		7.1 ± 6.4		5.4		61.9
		Nov–May		8.3 ± 7.8		8.0		61.9
		Jun–Oct		5.5 ± 3.2		3.5		36.7
PM_2.5–10_/PM_10_*a*		Overall		0.42 ± 0.18		0.25		0.93
		Nov–May		0.44 ± 0.20		0.31		0.93
		Jun–Oct		0.40 ± 0.13		0.18		0.81
CO (μg/m^3^)		Overall		281 ± 85		108		812
		Nov–May		300 ± 85		109		812
		Jun–Oct		254 ± 78		95		612
O_3_ (μg/m^3^)		Overall		60.0 ± 22.4		31.7		142.0
		Nov–May		57.8 ± 24.0		35.9		142.0
		Jun–Oct		63.1 ± 19.5		26.0		126.6
Temperature (°C)		Overall		7.7 ± 8.0		12.7		26.2
		Nov–May		2.8 ± 5.9		7.6		19.0
		Jun–Oct		14.4 ± 5.0		6.6		26.2
Relative humidity (%)		Overall		0.75 ± 0.13		0.20		0.99
		Nov–May		0.77 ± 0.14		0.20		0.99
		Jun–Oct		0.73 ± 0.12		0.17		0.97
**a**Fraction of PM_10_ that is PM_2.5–10_.								

**Figure 1:**
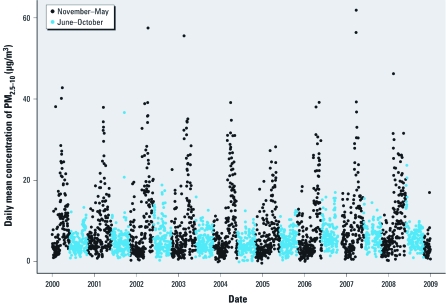
Seasonal variation in PM_2.5–10_ concentrations in Stockholm, Sweden, over the study period, 2000 through 2008.

Figure 1 shows that the daily mean concentrations of PM_2.5–10_ are highest during late winter and spring. For PM_2.5–10_, 148 days with a daily mean concentration of 20 μg/m^3^ (95th percentile) or higher were observed within the road dust period, but only 4 days outside that season (Figure 1). On average, PM_2.5–10_ is somewhat less than half (42%) of the total PM_10_ concentration. PM_2.5–10_ contributes 44% on average in November through May and 40% in June through October (Table 1).

The correlations between PM_2.5–10_ and the other pollutants are given in Table 2. We found a 1.68% increase [95% confidence interval (CI): 0.20%, 3.15%; *p* = 0.026] in daily mortality per 10-μg/m^3^ increase in PM_2.5–10_ (lag01) based on the single-pollutant model (Table 3). Adjusting for O_3_, PM_2.5_, or CO resulted in only minor decreases in effect estimates for PM_2.5–10_, with borderline significance (corresponding *p*-values = 0.06, 0.10, and 0.05, respectively).

**Table 2:** Correlation coefficients between variables in the study.

Pollutant	Season	PM_2.5_	PM_2.5–10_	CO	O_3_
PM_2.5_		Overall								
		Nov–May								
		Jun–Oct								
PM_2.5–10_		Overall		0.273						
		Nov–May		0.229						
		Jun–Oct		0.475						
CO		Overall		0.522		0.126				
		Nov–May		0.515		0.031				
		Jun–Oct		0.546		0.239				
O_3_		Overall		0.209		0.387		–0.192		
		Nov–May		0.187		0.478		–0.259		
		Jun–Oct		0.287		0.293		–0.003		
Temperature		Overall		0.050		–0.003		–0.257		0.435
		Nov–May		0.079		0.244		–0.172		0.441
		Jun–Oct		0.260		0.177		0.036		0.663
Relative humidity		Overall		–0.006		–0.418		0.278		–0.729
		Nov–May		–0.055		–0.569		0.248		–0.777
		Jun–Oct		0.054		–0.212		0.251		–0.630


**Table 3:** Mortality and PM_2.5–10_ association for lag01: overall estimates.

Model type	Pollutant	Percent increase per 10 μg/m^3^ (95% CI)	Percent increase per IQR (95% CI)*a*
Single pollutant						
PM_2.5–10_		PM_2.5–10_		1.68 (0.20, 3.15)		0.88 (0.11, 1.64)
PM_2.5_		PM_2.5_		1.46 (0.07, 2.84)		0.68 (0.03, 1.33)
Two pollutant						
PM_2.5–10_ + PM_2.5_		PM_2.5–10_		1.33 (–0.26, 2.92)		0.69 (–0.13, 1.52)
		PM_2.5_		0.90 (–0.62, 2.41)		0.42 (–0.29, 1.13)
PM_2.5–10_ + O_3_		PM_2.5–10_		1.47 (–0.07, 3.00)		0.77 (–0.04, 1.57)
		O_3_		0.31 (–0.32, 0.93)		0.94 (–0.98, 2.85)
PM_2.5–10_ + CO		PM_2.5–10_		1.53 (–0.02, 3.09)		0.80 (–0.01, 1.61)
		CO		0.04 (–0.09, 0.16)		0.37 (–0.87, 1.60)
All models adjusted for time trend, day of the week, public holidays, temperature, humidity, and influenza outbreaks. **a**IQR values: PM_2.5–10_, 5.2 μg/m^3^; PM_2.5_, 4.7 μg/m^3^; O_3_, 30.5 μg/m^3^; CO, 100 μg/m^3^.						

The effect estimate for a 10-μg/m^3^ increase in PM_2.5–10_ (1.33%; 95% CI: –0.26%, 2.92%; *p* = 0.10) was higher than the effect estimate for a 10-μg/m^3^ increase in PM_2.5_ (0.90%; 95% CI: –0.62%, 2.41%; *p* = 0.25) when both pollutants were included in the same model. In addition, the estimated percent change in daily mortality for an IQR increase was larger for PM_2.5–10_ (5.2 μg/m^3^) than for PM_2.5_ (4.7 μg/m^3^; Table 3).

**Figure 2:**
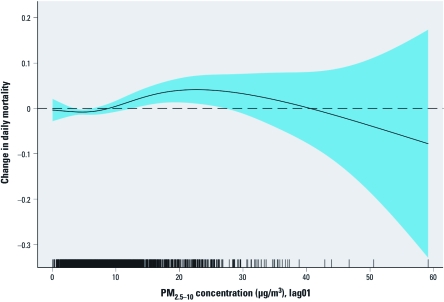
The smooth function of the relationship between PM_2.5–10_ (lag01) and daily mortality from the single-pollutant model, adjusted for time trend, day of the week, public holidays, temperature, humidity, and influenza outbreaks. The shaded area represents 95% CI.

The smooth function of PM_2.5–10_ (lag01)from the single-pollutant model, adjusted for the covariates listed above (Figure 2**)**, suggests that the more precisely estimated part of the curve does not deviate from linearity.

We estimated a 1.69% increase (95% CI: 0.21%, 3.17%; *p* = 0.025) in daily mortality per 10-μg/m^3^ increase in PM_2.5–10_ for the period November through May (Table 4). The effect estimate for the reference time period was lower (1.31%; 95% CI: –2.08%, 4.70%), but the difference between estimates was not statistically significant (*p* = 0.81).

**Table 4:** Mortality and PM_2.5–10_ association for lag01: by season.

Model type	Pollutant (season)	Percent increase per 10 μg/m^3^ (95% CI)	Percent increase per IQR (95% CI)*a*
Single pollutant						
PM_2.5–10_		PM_2.5–10_ (Nov–May)		1.69 (0.21, 3.17)		1.37 (0.17, 2.57)
		PM_2.5–10_ (Jun–Oct)		1.31 (–2.08, 4.70)		0.41 (–0.65, 1.46)
Two pollutant						
PM_2.5–10_ + PM_2.5_		PM_2.5–10_ (Nov–May)		1.38 (–0.21, 2.97)		1.12 (–0.17, 2.41)
		PM_2.5–10_ (Jun–Oct)		–0.28 (–5.06, 4.50)		–0.09 (–1.57, 1.40)
		PM_2.5_ (Nov–May)		0.72 (–0.92, 2.37)		0.37 (–0.48, 1.22)
		PM_2.5_ (Jun–Oct)		1.79 (–1.30, 4.88)		0.75 (–0.55, 2.05)
PM_2.5–10_ + O_3_		PM_2.5–10_ (Nov–May)		1.48 (–0.07, 3.02)		1.20 (–0.05, 2.45)
		PM_2.5–10_ (Jun–Oct)		1.26 (–2.13, 4.65)		0.39 (–0.66, 1.45)
		O_3_		0.30 (–0.33, 0.93)		0.92 (–1.01, 2.85)
PM_2.5–10_ + CO		PM_2.5–10_ (Nov–May)		1.55 (–0.01, 3.11)		1.26 (–0.01, 2.52)
		PM_2.5–10_ (Jun–Oct)		1.17 (–2.25, 4.59)		0.37 (–0.70, 1.43)
		CO		0.04 (–0.09, 0.16)		0.36 (–0.87, 1.59)
All models adjusted for time trend, day of the week, public holidays, temperature, humidity, and influenza outbreaks. **a**IQR values: PM_2.5–10_, 8.1 μg/m^3^ in November through May, 3.1 μg/m^3^ in June through October; PM_2.5_, 5.2 μg/m^3^ in November through May, 4.2 μg/m^3^ in June through October; O_3_, 30.5 μg/m^3^; CO, 100 μg/m^3^.						

After adjusting for other pollutants, there were only minor changes in the effect estimates for PM_2.5–10_ for the period November through May, and the magnitudes of the estimates were similar to the corresponding estimates for the whole year (Table 4).

Also, for the reference period June through October, changes in effect estimates were small after adjustments for CO and O_3_. However, no indication of an effect of PM_2.5–10_ was found when PM_2.5_ was adjusted for. The larger change in the estimated effect of PM_2.5–10_ may be explained by the larger effect estimate for PM_2.5_ and the higher correlation between PM_2.5_ and PM_2.5–10_ during the reference period (correlation coefficient *r* = 0.47) compared with the road dust period (*r* = 0.23; Table 2).

**Figure 3:**
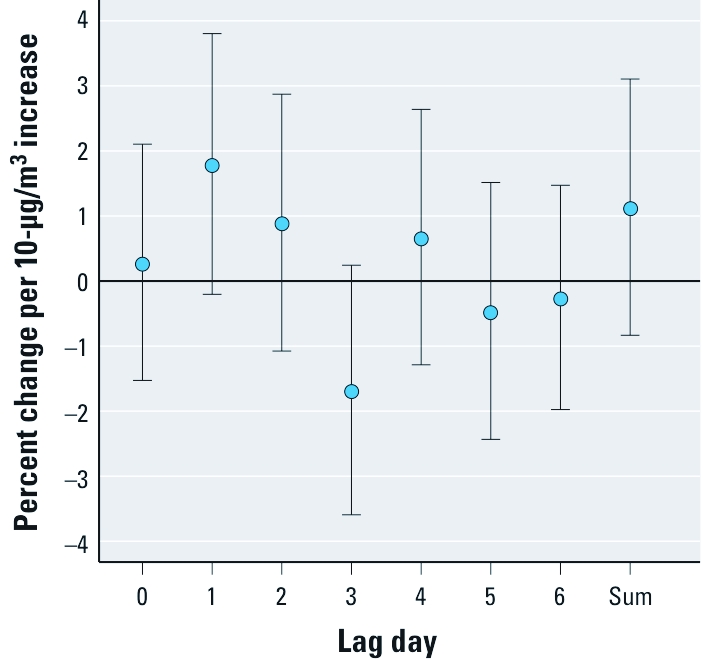
Percent change (95% CI) in daily mortality per 10-μg/m^3^ increase in PM_2.5–10_ for the 7-day distributed lag model.

When we examined the distributed lag model with 6 lag days for PM_2.5–10_, we found the largest coefficient for a 1-day lag and little evidence of mortality effects at longer lags (Figure 3). The association between mortality and the sum of the distributed lag (1.12%; 95% CI: –0.83%, 3.11%) was somewhat lower than the results for lag01 (1.68%; 95% CI: 0.20%, 3.15%).

## Discussion

The daily mean concentrations of PM_2.5–10_ are highest during late winter and spring, presumably due to increased suspension of road dust particles during dry road conditions (Ketzel et al. 2007; Omstedt et al. 2005). This is mainly because of the wear of stone materials in the asphalt by studded winter tires (Hussein et al. 2008; Omstedt et al. 2005).

We estimated a 1.7% increase in daily mortality per 10-μg/m^3^ increase in lag01 PM_2.5–10_, both for the whole year and during November through May, the high road dust period. This is a larger estimated effect than typically reported for PM_10_, for example, 0.6% (95% CI: 0.4%, 0.8%) per 10-μg/m^3^ increase in PM_10_ in the European APHEA 2 study (Katsouyanni et al. 2001). The effect estimates for O_3_ and CO were similar to those reported for APHEA 2 (Gryparis et al. 2004; Samoli et al. 2007).

When data were split into two different time periods, the estimated effect associated with PM_2.5–10_ was higher during November through May, the road dust period, consistent with recent findings reported for PM_2.5–10_ and PM_10_ that indicated stronger associations during springtime (Zanobetti and Schwartz 2009; Zeka et al. 2006). Seasonal variation in associations could reflect greater indoor penetration during months when windows are open (Zeka et al. 2006) or seasonal variation in the composition of PM from different sources. In our case, the seasonal difference is not likely explained by indoor penetration, because associations were stronger during the winter, when windows are likely to be closed. However, the chemical composition is likely important, because PM produced during the winter period is dominated by stone minerals from road dust.

Detailed chemical analyses of sampled PM_2.5–10_ during that period have shown that the PM is dominated by quartzite, which was the most common stone mineral in the pavements in Stockholm (Furusjö et al. 2007; Sjödin et al. 2010).

We did not directly monitor PM_2.5–10_ but estimated its concentration as the difference between PM_10_ and PM_2.5_. This means that part of the variability in the concentration of PM_2.5–10_ is due to the measurement errors in both PM_10_ and PM_2.5_. Comparison with a gravimetric method has shown that the relative uncertainty of the determination of PM_10_ [according to the EU guidance for demonstrating equivalence of ambient air monitoring methods (European Commission Working Group on Particulate Matter 2002)] in Stockholm using the TEOM instrument is between 11% and 27% at the daily limit value (50 μg/m^3^). The uncertainty of PM_2.5–10_ has not been determined, but most of the uncertainty in PM_10_ is likely due to the correction for volatile PM, which is mainly present in PM_2.5_ fraction and not in the PM_2.5–10_ fraction.

A strength of the present study is that PM_2.5–10_ during the road dust period originates from the road network covering the whole city, not from a few point sources. Thus, in Stockholm, where the main contributor to PM_2.5–10_ is road traffic, the urban background daily mean concentrations in different parts of the city should fluctuate in a similar way.

The present study and other recent studies for which exposure can be expected to be spatially homogeneous support the hypothesis that there is a short-term effect on mortality, and it is at least as strong as typically found for PM_10_. Other recent studies also have estimated large effects on daily mortality of crustal PM_2.5–10_ (Malig and Ostro 2009; Perez et al. 2008; Zanobetti and Schwartz 2009).

There is support for cardiopulmonary effects of PM_2.5–10_ from recent human experimental studies. When 14 healthy young volunteers were exposed to concentrated ambient PM_2.5–10_ (2 hr; mean, 89 μg/m^3^) and filtered air in a single-blind, crossover study, exposure produced a mild pulmonary inflammation (Graff et al. 2009) 20 hr after exposure; a decrease in blood tissue plasminogen activator, which is involved in fibrinolysis; and a decrease in heart rate variability, which indicates an effect on the autonomic nervous system. Reduced heart rate variability is a prognostic marker for the development of cardiac arrhythmias (van Boven et al. 1998).

In a Swedish experimental study, Gustafsson et al. (2008) generated particles from the wear of studded tires on two pavements and traction sanding using a road simulator. A chemical analysis showed that the generated wear particles consisted almost entirely of minerals from the pavement stone material. It is well known that silica, which is part of this stone material, is capable of producing reactive oxygen species, either directly on the particle surface or by cells in response to exposure (Hamilton et al. 2008). In a recent study, Karlsson et al. (2011) examined the toxicoproteomic effects on human monocyte-derived macrophages after exposure to wear particles generated from the interface of studded tires and a granite-containing pavement. They showed that overall, proteins associated with inflammatory response were increased and proteins involved in cellular functions such as redox balance, antiinflammatory response, and glycolysis were decreased. Activation of the inflammatory pathway is one potential explanation for cardiopulmonary effects associated with exposure to mineral particles.

Road dust is an important traffic-related pollutant, because road wear particles contributes substantially to local particle emissions in cities. In cities where studded tires are used, road dust may cause violations of limit values for PM_10_. The effect of PM_2.5–10_ on mortality has been questioned because of many inconsistent findings when controlling for PM_2.5_ (Brunekreef and Forsberg 2005). This has influenced discussions on limit values and abatement strategies. Several recent studies (Malig and Ostro 2009; Perez et al. 2008; Samoli et al. 2011; Zanobetti and Schwartz 2009; Zauli Sajani et al. 2010) have, like the present one, produced evidence of a short-term effect of PM_2.5–10_ and crustal PM_10_ (not originating from combustion processes) on mortality. Results regarding the effect modification of Saharan dust days on a PM_10_–mortality relationship are inconsistent, despite positive associations, with negative effects and no interaction effects also reported. These inconsistent findings could reflect differences in the composition of other PM_10_ fractions, but also differences in correlations with other pollutants.

## Conclusions

Given our results on road dust and other recent findings showing an impact of PM_2.5–10_ on daily mortality in studies of U.S. cities (Malig and Ostro 2009; Zanobetti and Schwartz 2009) and desert dust on daily mortality in Barcelona (Perez et al. 2008), it seems appropriate to separately regulate and control PM_2.5–10_. One must keep in mind that a large proportion of PM_2.5_ in many cities is transported over long distances and is difficult to avoid, whereas PM_2.5–10_, as in Stockholm, may be largely of local origin. Therefore, it also may be easier to improve health by reducing exposures to PM_2.5–10_.
